# Primary ameloblastic carcinoma of the maxilla: A case report and literature review

**DOI:** 10.3892/ol.2014.2654

**Published:** 2014-10-31

**Authors:** NARIKAZU UZAWA, MIHO SUZUKI, CHIKA MIURA, NOBUYOSHI TOMOMATSU, TOSHIYUKI IZUMO, KIYOSHI HARADA

**Affiliations:** 1 Maxillofacial Surgery, Department of Maxillofacial Reconstruction and Function, Division of Maxillofacial and Neck Reconstruction, Graduate School, Tokyo Medical and Dental University, Tokyo 113-8549, Japan; 2 Diagnostic Oral Pathology, Division of Oral Health Science, Graduate School, Tokyo Medical and Dental University, Tokyo 113-8549, Japan

**Keywords:** ameloblastic carcinoma, maxilla, odontogenic, primary

## Abstract

Ameloblastic carcinoma (AC) is a rare malignant odontogenic neoplasm that tends to occur in the mandible rather than in the maxilla. This malignancy is classified as a tumor that combines the morphological features of ameloblastoma and carcinoma, regardless of the presence or absence of metastasis. In addition, AC has been classified into two types, primary and secondary. The former develops *de novo* and the latter develops by malignant transformation of a pre-existing benign ameloblastoma. The present study describes the case of a 22-year-old patient with primary AC of the maxilla. A review of the literature focusing on the clinical details, treatment results and histopathological and phenotypic information available for ameloblastic carcinoma of the maxilla from a 60-year period was also performed. As a result, it was found that primary AC is dominant in the maxilla and does not exhibit an aggressive phenotype compared with secondary AC. In addition, the presence of recurrence was found to correlate with mortality, indicating that early, aggressive and complete removal of the tumor is the best treatment for survival.

## Introduction

The most common benign odontogenic tumor of the jaw is ameloblastoma, whereas ameloblastic carcinoma (AC) is rare. For a long time, malignancy in ameloblastoma has been the subject of controversy (1,2). In the 2005 World Health Organization (WHO) classification (3), odontogenic carcinomas included malignant ameloblastoma, AC and primary intraosseous, ghost cell odontogenic and clear cell odontogenic carcinomas. Malignant ameloblastoma is described as a metastasizing ameloblastoma that presents with benign histological characteristics in primary and metastatic lesions. By contrast, AC is considered to be a rare malignant odontogenic tumor that has combined histopathological features of ameloblastoma and carcinoma, regardless of the presence or absence of metastasis. Furthermore, AC has been classified into two types, primary and secondary. The former develops *de novo* and the latter develops by malignant transformation of a pre-existing benign ameloblastoma (3).

The mean age of AC occurrence is 30.1 years, but a wide range of ages can be affected. There is no proven gender bias, but certain studies have reported a male predominance (4,5). Similar to ameloblastoma, AC is commonly located in the posterior portion of the mandible and is extremely rare in the maxillary region. The most usual clinical complaint is swelling, but other symptoms, including dysphonia, associated pain, trismus and rapid growth have been reported (2,4). Radiography of AC can reveal poorly-defined radiolucency, occasionally with focal radiopacities. These findings, which are extremely unusual for ameloblastoma, could be due to necrosis with dystrophic calcification in AC (4,6). With regard to clinical behavior, AC tends to be aggressive and extends with local destruction. Lymph node involvement and distant metastasis to various regions have also been reported (4,7). Therefore, diagnostic imaging prior to treatment is extremely important. In comparison to AC of the mandible, AC of the maxilla has not yet been well documented due to the lack of information about this rare carcinoma.

The present study reports the clinical, histological, immunohistochemical and therapeutic details of a case of maxillary AC with a 22-month follow-up period. In addition, the present study reviews a 60-year period of the literature with regard to the clinical details, treatment results and histopathological and phenotypic information available for AC of the maxilla. Written informed consent was obtained from the patient.

## Case report

### Patient characteristics and case presentation

**Figure 1:**
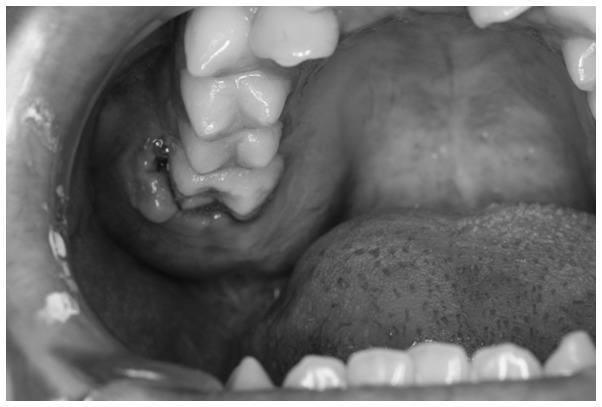
Clinical examination. Intraoral image revealing a mass with an elastic, hard, well-defined swelling and a smooth surface in the right maxillary molar region. The lesion measured 31×25×15 mm in size.

A 22-year-old male was referred to the Department of Oral and Maxillofacial Surgery (Graduate School, Tokyo Medical and Dental University Hospital, Tokyo, Japan). The patient complained of painless swelling in the right maxilla that had been present for one month. The facial configuration appeared symmetrical upon clinical examination, but an intraoral examination revealed elastic, hard, well-defined swelling with a smooth surface in the right maxillary molar region. The lesion measured 31×25×15 mm in size (Fig. 1).

**Figure 2:**
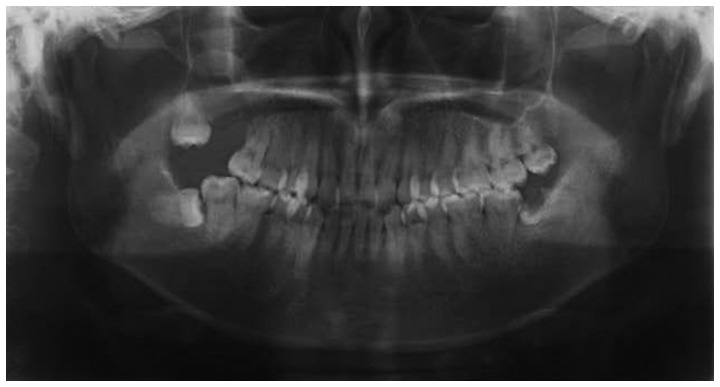
Panoramic radiographic finding. Panoramic radiograph revealing the cystic radiolucent lesion in the right maxilla elevating the floor of the right maxillary sinus, indicating the presence of a cystic lesion or odontogenic tumor of the right maxilla.

**Figure 3:**
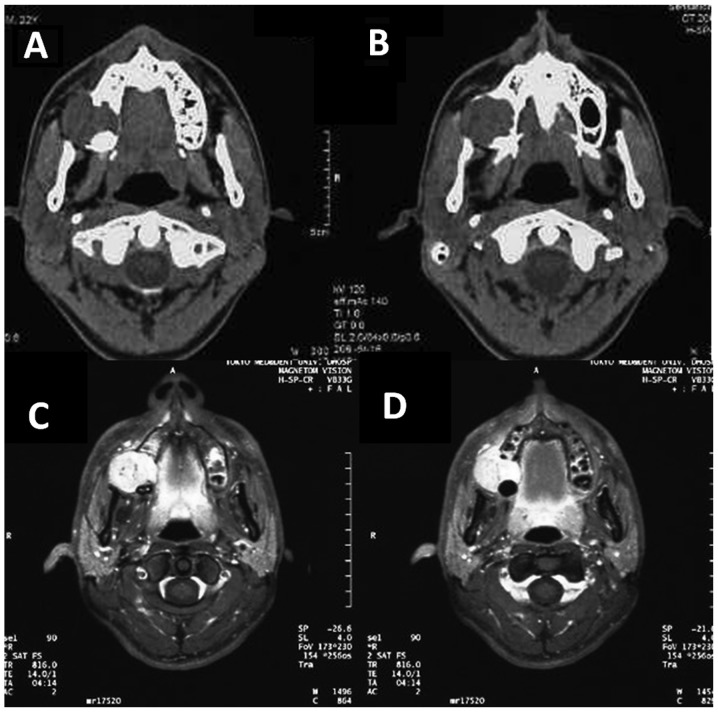
(A and B) Computed tomography (CT) and (C and D) Axial contrast-enhanced magnetic resonance imaging (CE-MRI). (A) Axial CT image revealing a globular-shaped lesion arising from the inside maxillary bone with destruction of its posterior wall and alveolar bone. (B) The lesion exhibited intermediate signal intensity on T1-weighted imaging, and heterogeneous high signal intensity on T2-weighted and short TI inversion-recovery imaging. (C and D) CE-MRI revealed a distinctly-bordered lesion (31×30 mm) extending from the right maxillary alveolar process to the right palate and reaching the retromaxillary fat space.

Panoramic radiography revealed a cystic radiolucent lesion in the right maxilla elevating the floor of the right maxillary sinus (Fig. 2). Computed tomography (CT) examination was subsequently performed. The axial CT image revealed a globular-shaped lesion arising from the inside of the maxillary bone, with destruction of the posterior wall and alveolar bone. The diameter of this lesion reached 30 mm in size. The right maxillary tuberosity and pterygoid plates appeared to be intact, but coronal CT imaging revealed destruction of the elevated sinus floor in the right posterior maxilla. The margin of the lesion was almost well defined. These findings indicated that this lesion was a benign tumor, such as an ameloblastoma (Fig. 3A and B). An incision biopsy was performed and the lesion was revealed not to be cystic, but to be a solid mass. Although the biopsy revealed that the lesion was an odontogenic carcinoma, its histopathological type was unidentifiable. Next, ^18^F-fluorodeoxyglucose-positron emission tomography (FDG-PET)/CT was performed to examine the extent of the primary lesion and the presence of regional lymph node and distant metastasis. Furthermore, contrast-enhanced magnetic resonance imaging (CE-MRI) with gadodiamide, including a dynamic study, was performed to evaluate soft-tissue invasion at the maxillary sinus and pterygopalatine fossa.

**Figure 4:**
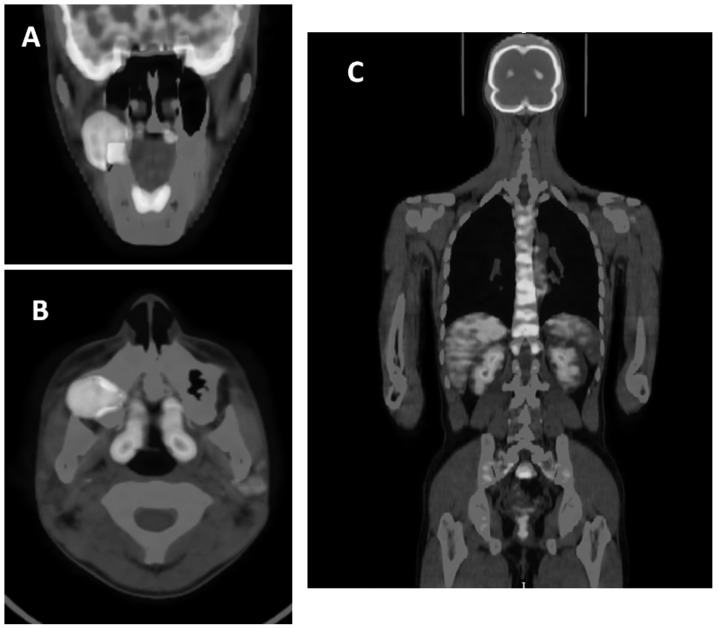
FDG-PET images. (A) axial and (B) coronal FDG-PET/CT images revealing a slight FDG uptake in the primary tumor of the right maxilla and bilateral superior internal jugular nodes. (C) No abnormal uptake, which would indicate distant metastasis, was observed on FDG-PET images. FDG-PET, ^18^F-fluorodeoxyglucose-positron emission tomography; CT, computed tomography.

On FDG-PET imaging, slightly elevated FDG uptake was identified in the right maxilla and bilateral superior internal jugular nodes. The maximum standardized uptake value (SUV_max_) was 5.6 in the right maxilla, 3.3 in the right cervical lymph node and 2.6 in the left cervical lymph node. No abnormal uptake indicating distant metastasis was observed on FDG-PET images (Fig. 4). CE-MRI revealed a distinctly-bordered lesion that was 31×30 mm in size and extended from the right maxillary alveolar process to the right palate and reached the retromaxillary fat space. This lesion exhibited intermediate signal intensity on T1-weighted imaging and heterogeneous high signal intensity on T2-weighted and short TI inversion-recovery imaging (Fig. 3C and D). In addition, ultrasonography was performed to evaluate the bilateral superior internal jugular nodes, which exhibited slight FDG uptake on the FDG/PET analysis. The findings did not indicate that a metastatic lymph node lesion was present.

On the basis of these imaging findings, the patient was diagnosed with an odontogenic carcinoma of the right maxilla (T4N0M0, stage IV). The patient underwent a right partial maxillectomy and full-thickness skin grafting from the left inguinal region. Following the surgery, the diagnosis was histopathologically confirmed using the whole surgical specimen. These lesions were pathologically diagnosed as AC.

**Figure 5:**
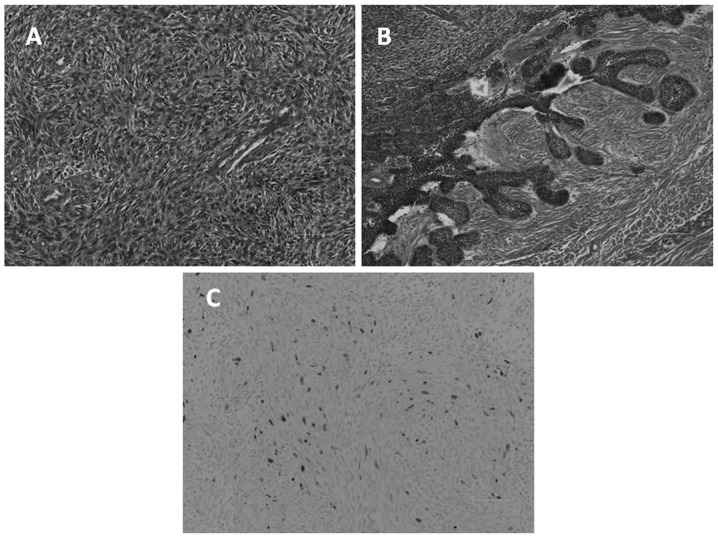
Microscopic examination. (A) The majority of the mass consisted of spindle tumor cells exhibiting a storiform, pseudosarcomatous pattern. The epithelial component demonstrated cytological malignancy, characterized by nuclear pleomorphism, an increased nucleus to cytoplasm ratio, hyperchromatic nuclei and a high mitotic rate. (B) In the other area, the tumor cell nest exhibited peripheral palisading of columnar cells, with a vacuolated cytoplasm and reverse-polarized nuclei. These findings resemble those for ameloblastoma. (C) The Ki-67 proliferation index was 5%, indicating that this tumor was of low malignancy.

Microscopic examination revealed the presence of an osteolytic mass with slit-like cystic formation. The majority of the mass consisted of spindle tumor cells exhibiting a storiform, pseudosarcomatous pattern. The epithelial component demonstrated cytological malignancy, characterized by nuclear pleomorphism, an increased nucleus to cytoplasm ratio, hyperchromatic nuclei and a high mitotic rate (Fig. 5A). By contrast, an alternative region of the tumor, the tumor cell nest, revealed peripheral palisading of columnar cells, with a vacuolated cytoplasm and reverse-polarized nuclei. These findings were similar to those for ameloblastoma (Fig. 5B). In the immunohistological assessment, the specimen was found to be positive for cytokeratin AE1/3 and vimentin expression. The Ki-67 proliferation index was 5%, indicating that the tumor was of low malignancy (Fig. 5C). Therefore, it was concluded that the tumor was a primary AC, based on the histopathological and immunohistochemical findings.

The post-operative progress of the patient was fair, resulting in discharge from the hospital on the 22nd day. At the 22 months post-surgical follow-up examination, the patient was free of symptoms and neither recurrence nor metastases were detected.

### Analysis of the literature on AC, including the present case

**Figure 6:**
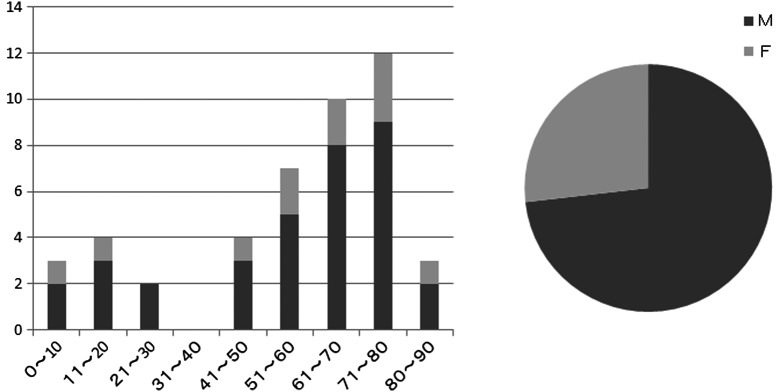
Age and gender. Age and gender distribution revealing the occurrence of maxillary ameloblastic carcinoma in different age groups and genders.

A review of the English literature published between 1948 and 2012 revealed 45 cases of maxillary AC, including the present case (1,2,4–33). These cases are summarized in Table I. The 45 patients ranged in age between 5 and 90 years, with an average age of 55.2 years. A breakdown of the age distribution is presented in Fig. 6. The studies reported the cases of 34 males and 11 females, with a male to female ratio of 3:1. The predominant symptom of AC was swelling, followed by ulceration, pain and bleeding. According to the literature, AC occurs most often in the posterior maxilla.

The type of AC was classified according to the clinical, follow-up, histopathological and phenotypic information available for these cases. As a result, 27 cases (60%), including the present case, developed *de novo*, primary AC, and 13 cases (29%) arose from a pre-existing ameloblastoma as secondary AC. The remaining three cases could not be determined using the information provided and two cases presented with a benign histological appearance in the primary and metastatic regions, indicating malignant ameloblastoma. Of the 27 primary and 13 secondary AC cases, follow-up data were available for 23 primary cases and 10 cases of carcinoma ex ameloblastoma. Of the 23 cases with both primary AC and follow-up data, only three patients (13.0%) succumbed to the disease. By contrast, four of the 10 cases of secondary AC with follow-up data (40%) succumbed to the tumor.

With regard to the first treatment modality for the primary lesions, 28 of the 45 cases (62.2%) only underwent surgical resection and 14 (31.1%) underwent surgical resection and adjuvant radiotherapy. In the remaining three cases, biopsy only was performed in two cases and palliative tumor reduction was performed in one case. When these six cases were excluded, primary recurrence occurred in 15 of the 39 cases (38.5%). Although eight out of the 15 patients (53.3%) experienced recurrence only once, the remaining seven patients experienced recurrence several times. The mean duration between the primary treatment and the initial recurrence was 47.5 months, with a wide range of 3–151 months. In addition, in seven of the 15 primary recurrence cases (46.7%), distant metastasis was observed in several regions. In total, 10 of the 39 patients (25.6%) experienced metastatic lesions. Regional metastasis occurred in three cases and distant metastasis occurred in nine cases. In the cases with regional metastasis, two involved lymph node metastasis and the remaining case involved maxillary AC that had metastasized to the mandible. The most common region of distant metastasis was the lung, occurring in nine cases (8–12,15,25,27,33). Distant metastasis was also reported in the liver in two cases (27,32), in the bone in two cases (9,25), in the brain in one case (32) and in the myocardium in one case (25).

**Figure 7:**
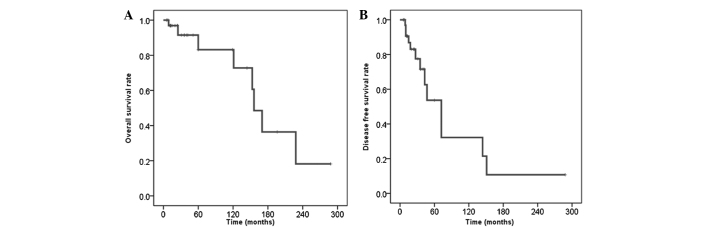
Kaplan-Meier curves for (A) disease-free survival (DFS) and (B) overall survival (OS). The five-year and 10-year DFS rates were 53.7 and 32.2%, respectively. The five-year OS rate was 83.2% and the 10-year rate was 32.2%, the same as the DFS rate.

Survival analyses were performed on the 35 cases with follow-up data. Three cases not undergoing curative treatment and seven cases without a description of the treatment outcome were excluded from the survival analyses. Overall, 12 of the 35 patients (34.3%) had experienced a recurrence of the disease and eight patients (22.9%) succumbed to AC. Kaplan-Meier survival curves for disease-free survival (DFS) and overall survival (OS) are presented in Fig. 7A and B, respectively. The five- and 10-year DFS rates were 53.7 and 32.2%, respectively. The five-year OS rate was 83.2% and the 10-year OS rate was 32.2%, the same as that for DFS. Although approximately half of the cases experienced recurrence of the disease in less than five years, salvage treatment appeared to be successful in several cases.

## Discussion

There has been controversy regarding the definition and classification of AC in the past. The 1972 WHO classification of odontogenic carcinoma included malignant ameloblastoma, but the term AC was not used in that classification. The term malignant ameloblastoma refers to tumors that metastasize to several regions while the histological appearance of the primary and metastatic lesions remains benign (34). The term AC was introduced by Elazy in 1982 (35). In addition, in 1984, Slootweg and Müller provided definitions and nomenclature used to distinguish AC from malignant ameloblastoma (36). In the 2005 WHO classification, AC is defined as a rare odontogenic malignant tumor in which the histopathological features of ameloblastoma and malignancy coexist. In addition, AC can develop *de novo*, as the primary type, or by malignant transformation of an ameloblastoma, as the secondary type, with a distinction between carcinoma ex intraosseous ameloblastoma and carcinoma ex peripheral ameloblastoma (3). In the present study, no pre-existing ameloblastoma in the right side of the maxilla was identified and the presence of the combined histopathological features of ameloblastoma and malignancy were confirmed. Therefore, the tumor in the present case was diagnosed as a primary AC.

Recently, Casaroto *et al* reported a case of AC that arose in the mandible and also presented a literature review of AC classified into primary or secondary types using the recent WHO classification (40). In total, 31 studies published between 2005 and 2011 were reviewed, with 15 cases arising from the maxilla and 16 from the mandible. It was indicated that the primary type occurs more frequently in the maxilla, unlike the secondary type, which was reported more often in the mandible. In addition, it was found that the secondary type appears to correlate with recurrence and mortality, suggesting that it is more aggressive compared with the primary type. The present study also reviewed 45 AC cases that had occurred in the maxilla from a 60-year period. The results of the present study were compatible with those of the aforementioned study and confirmed that primary AC is dominant in the maxilla and is not as aggressive as secondary AC.

Due to the rarity of large clinical series and long-term follow-up, there is no consensus on the treatment of AC. Based on follow-up data from the present review, radical surgical resection appears to be the most reliable treatment of choice. In the present review, wide surgical resection was performed in 42 of the 45 cases (93.3%). With regard to the surgical margin, Avon *et al* advocated 2- or 3-cm bony margins for an en bloc removal (23). In addition, Zwahlen and Grätz also recommended partial maxillectomy with a 10–15-mm safety margin of healthy bone, including the lateral nasal wall, alveolar ridge, mucosa of the maxillary sinus and hard palate (41). However, even if various ACs occurred in the same patient, it was revealed that aggressiveness varied according to whether the AC was primary or secondary (40). Thus, surgical margins should be determined with consideration of tumor types. In the cases of secondary AC, the surgical margin should be set to at least 10–15 mm. By contrast, in the case of primary AC, it may be possible to decrease the surgical margins. Neck dissection should also be considered when there is evident lymphadenopathy. By contrast, controversy remains regarding the treatment of AC, with certain studies suggesting radiotherapy (26) and others doubting its effectiveness (27). Although primary radiotherapy is not a reliable treatment modality, it is expected to be useful in cases with perineural or massive soft-tissue invasion and in cases with positive surgical margins (26). In the present review, radiotherapy was used as either a primary or secondary treatment in the 20 cases (44.4%) with metastatic or recurrent disease out of the 45 total cases. Experience with chemotherapy as a treatment of AC is minimal. In the present study, only three patients with a progressive AC were treated with chemotherapy. One of these patients succumbed to AC and the response to this treatment was not described in the remaining cases. Several studies have also reported that this modality appears to have limited value in the treatment of AC (37,38).

In the present study, the local recurrence of AC occurred in 15 out of 39 cases (38.4%). In addition, half of these cases experienced recurrence several times and distant metastases occurred in several regions. The presence of recurrence appears to correlate with mortality, since the majority of the cases that resulted in mortality had a previous history of tumor recurrence. These findings strongly indicate that an early, aggressive and complete removal of the tumor is the best treatment for survival. Additionally, a more radical and aggressive treatment modality is required in cases with primary recurrence. The other significant problem in treating AC is that the period of recurrence and distant metastasis is long compared with other malignancies that occur in the head and neck regions, such as squamous cell carcinomas. In the present review, the average period between primary treatment and recurrence was 47.5 months, with a wide range of 3 to 151 months. In addition, the mean interval between the initial treatment and the manifestation of distant metastasis was 84.7 months, although the development of metastasis reached up to 156 months after primary treatment. Since there is no definitive modality or strategy for a follow-up of this tumor, long-term periodic follow-up following surgical resection is indispensable for the early detection of recurrence and metastatic lesions.

The nuclear protein Ki-67 antigen has been used to determine the proliferation rate of numerous types of tumors and cystic lesions. This is a reliable marker of cellular proliferation. The results on immunohistochemistry for the Ki-67 labeling index (LI) in seven maxillary ACs, including the present case, are as follows: Yoon *et al* reported six cases of AC, with five cases occurring in the maxilla and one case occurring in the mandible, and the mean Ki-67 LI of these six cases was determined to be 13.91% (standard deviation, 6.96; range, 9.30–22.9%) (2). Yazici *et al* also examined a case of maxillary AC that occurred in a 10-year-old male, and the Ki-67 LI was determined to be 10% (29). In the present case, immunohistochemical examination of Ki-67 was performed on the only biopsy specimen, but the LI was only 5%. This result suggests that the tumor in the present case possessed low malignancy compared with those in the previous studies.

AC is known to have not only locally invasive features, but to also result in regional and distant metastases. AC metastasizes to the lung and other regions, including the cervical lymph nodes, brain, bones, soft tissue and liver. Thus, the extension of the lesion must be closely assessed and the patient must be carefully examined to exclude the existence of metastases and lesions elsewhere in the body. FDG-PET is a useful modality for the evaluation of malignant tumors in the primary site and the detection of regional lymph node and distant metastasis. However, there have been a few studies investigating FDG-PET of AC. Matsuzaki *et al* previously reported a case of maxillary AC where strong FDG uptake (SUV_max_, 28.3) was observed in the primary tumor. However, there were no abnormal FDG accumulations that suggested metastasis in that case (31). In the present case, slightly elevated FDG uptake was observed in the primary lesion (SUV_max_, 5.6) and bilateral superior internal jugular nodes (right side SUV_max_, 3.3; left side SUV_max_, 2.6). No abnormal uptake that would suggest distant metastasis was observed on the FDG-PET images in the present case. Since AC has the potential for distant metastasis, with or without cervical lymph node metastasis, it is essential to use PET for the initial whole-body examination prior to surgery.

In summary, the present study reports the case of a 22-year-old male patient with AC of the maxilla. AC is rare disorder and its treatment remains controversial. The prognosis of AC is dominated by the risk of local recurrence and distant metastases, but the present patient has not yet experienced recurrence or metastasis during the 22-month post-surgical follow-up. Continued and long-term follow-up is mandatory to detect late recurrence and metastasis. In addition, continued research, case studies and treatment experience are necessary to establish more useful treatment and management strategies for this rare tumor.

## Figures and Tables

**Table I:** Axillary ameloblastic carcinomas: Review of published reports.

Case	First author, year (ref.)	Gender	Age	Symptoms	Location	Tumor type	Primary treatment	Time between treatment and recurrence, months	Treatment for recurrence	Time to metastasis, months	Site of metastasis	Follow-up, months	Outcome
1	Grimes and Stephens, 1948 (8)	F	56	Unknown	Po	S	S/R			120	Lung	120	NM
2	Eda *et al*, 1972 (9)	F	44	Painless swelling	Po	S	S^a^	1st, 43	S^a^	120	LN, lung, vertebra	121	Dc
								2nd, 32	S^a^				
								3rd, 8	S^a^				
								4th, 8	S^a^				
								5th, 5	R				
3	Krempien *et al*, 1979 (10)	M	5	Unknown	NM	M	S^a^			72	LN, lung	144	Ao
4	Daramola *et al*, 1980 (11)	M	22	Swelling	Po	S	S^a^	1st, 24	S^a^	60	Lung	NM	NM
								2nd, 36	C/R				
5	Madiedo *et al*, 1981 (12)	M	49	Swelling	Po	S	S^a^	1st, 18	S+ND/R	36	Lung	60	Dc
								2nd, 42	C				
6	Andersen and Bang, 1986 (13)	M	77	Bleeding	S	S	S^a^	36	S^a^			NM	NM
7	Nadimin *et al*, 1986 (14)	F	15	Swelling	APo	P	S^a^					NM	NM
8	Corio *et al*, 1987 (4)	M	15	Swelling	NM	ND	S^a^					NM	NM
9	Inoue *et al*, 1988 (15)	F	51	Swelling	Po	S	S^a^	137	S^a^	145	Lung	186	Dc
10	MacClatchey *et al*, 1989 (16)	F	77	Concavity, granulation	Po	P	S^a^					24	Ao
11	Lee *et al*, 1990 (17)	M	56	Pain	Po	P	S/R	3	Untreated	6	Mandible	7	NM
12	Lolachi *et al*, 1995 (18)	F	82	Trismus, bleeding	S	P	S^a^					NM	NM
13	Ingram *et al*, 1996 (19)	M	83	Pain, erosion	Po	P	S/R					24	Ao
14	Infante-Cossio *et al*, 1998 (20)	F	69	Painless swelling, anaethesia	APoS	P	S/R					60	Ao
15		M	77	Swelling, pain, anaesthesia	APoS	P	S/R		Untreated			9	Dc
16		M	64	Swelling, fistula	PoS	P	S/R					36	Ao
17	Sastre *et al*, 2002 (21)	M	40	Painful swelling	A	P	S/S					24	Ao
18	Dhir *et al*, 2003 (22)	M	72	Unknown	PoS	P	S/R					20	Ao
19	Avon *et al*, 2003 (23)	M	68	Fistula	PoMS	P	S/S					24	Ao
20	Oginni *et al*, 2003 (24)	F	61	Bleeding	Po	P	S^a^	15	Untreated			15	Ac
21	Zwahlen *et al*, 2003 (25)	M	44	Ulcer	Po	M	S/R	1st, 72	S^a^	156	Lung, mycardial, skull base	156	Dc
								2nd, 24	S^a^				
								3rd, 12	R/S^b^				
22	Goldenberg *et al*, 2004 (7)	M	72	Unknown	NM	ND	S/R					36	Ao
23	Philip *et al*, 2005 (26)	M	70	Unknown	NM	P	S/R					40	Ao
24		M	56	Unknown	NM	P	S/R					8	Ao
25	Hall *et al*, 2007 (27)	M	15	Swelling	A	S	S^a^	1st, 10	S^a^			196	Ao
								2nd, 28	S^a^				
								3rd, 2	S^a^				
26		M	16	Swelling	PoMS	S	S^a^					288	Ao
27		M	75	Numbness, loose tooth, nasal obstruction	PoMS	S	S^a^	27	S^a^			153	Do
28		F	7	Swelling	Po	S	S^a^	35	S^a^			119	Ao
29		M	63	Swelling, ulcer	PoMS	S	S^a^	1st, 151	S^a^			228	Dc
								2nd, 13	S^a^				
								3rd, 50	S^a^				
								4th, 14	Biopsy				
30		M	52	Nasal congestion, pain	PoMS	S	S^a^	47	C	47	Lung, liver	51	Ac
31	Ward *et al*, 2007 (28)	M	64	Swelling, erythema	A	P	S^a^					42	Ao
32	Benlyazid *et al*, 2007 (5)	M	90	Exophytic, ulcer	Po	P	S^a^					25	Do
33	Naik and Kale, 2007 (6)	M	70	Swelling	APoMS	P	S^a^					12	Ao
34	Yazici *et al*, 2008 (29)	M	10	Swelling	S	P	S/R					6	Ao
35	Angiero *et al*, 2008 (1)	M	68	Bleeding	PoMS	S	S^a^					6	Ao
36	Yoon *et al*, 2009 (2)	M	63	Ulcer, swelling	Po	P	S/R	1st, Unknown	S^a^			13	Ao
								2nd, Unknown	S^a^				
37		F	73	Pain, swelling	PoMS	P	S^a^					31	Ao
38		M	61	Pain, swelling, trismus	PoMS	P	Biopsy					NM	NM
39	Yoon *et al*, 2009 (2)	M	58	Pain, ulcer	P	P	S+ND					12	Ao
40	Lucca *et al*, 2010 (30)	M	73	Swelling	APoMS	P	Biopsy					4	Dc
41		M	69	Ulcer	P	P	S^a^					11	Ao
42	Matsuzaki *et al*, 2011 (31)	F	73	Swelling	PoMS	P	S^a^					12	Ao
43	Nicolotti *et al*, 2011 (32)	M	77	Swelling, ulcer	AP	P	S^b^			0	Lung, liver, cerebal	5	Dc
44	França *et al*, 2012 (33)	M	59	Swelling, pain	APoMS	ND	S/R					24	Ao
45	Present case	M	22	Swelling	Po	P	S^a^					13	Ao

Po, posterior (distal to canine); A, anterior (incisor to canine); MS, involvement of maxillary sinus; NM, not mentioned; P, primary type; S, secondary type; M, malignant amleoblastoma; ND, not determined; S^a^, surgery alone; S/R, surgery with adjuvant radiotherapy; ND, neck dissection; S^b^, palliative tumor reduction; C, chemotherapy; Ao, alive without cancer; Ac, alive with cancer; Do, mortality due to other cause; Dc, mortality due to disease; NM, not mentioned.
